# Analgesic Activity of Acid-Sensing Ion Channel 3 (ASIС3) Inhibitors: Sea Anemones Peptides Ugr9-1 and APETx2 versus Low Molecular Weight Compounds

**DOI:** 10.3390/md16120500

**Published:** 2018-12-12

**Authors:** Yaroslav A. Andreev, Dmitry I. Osmakov, Sergey G. Koshelev, Ekaterina E. Maleeva, Yulia A. Logashina, Victor A. Palikov, Yulia A. Palikova, Igor A. Dyachenko, Sergey A. Kozlov

**Affiliations:** 1 Shemyakin-Ovchinnikov Institute of Bioorganic Chemistry, Russian Academy of Sciences, ul. Miklukho-Maklaya 16/10, 117997 Moscow, Russia; yaroslav.andreev@yahoo.com (Y.A.A.); osmadim@gmail.com (D.I.O.); sknew@yandex.ru (S.G.K.); katerina@1ns.ru (E.E.M.); yulia.logashina@gmail.com (Y.A.L.); 2 Institute of Molecular Medicine, Sechenov First Moscow State Medical University, Trubetskaya str. 8, bld. 2, Moscow 119991, Russia; 3 Branch of the Shemyakin-Ovchinnikov Institute of Bioorganic Chemistry, Russian Academy of Sciences, 6 Nauki Avenue, 142290 Pushchino, Russia; viktorpalikov@mail.ru (V.A.P.); ulia2791@rambler.ru (Y.A.P.); dyachenko@bibch.ru (I.A.D.)

**Keywords:** acid-sensing ion channel, animal models, pain relief, toxin, Ugr 9-1, APETx2

## Abstract

Acid-sensing ion channel 3 (ASIC3) makes an important contribution to the development and maintenance of inflammatory and acid-induced pain. We compared different ASIC3 inhibitors (peptides from sea anemones (APETx2 and Ugr9-1) and nonpeptide molecules (sevanol and diclofenac)) in anti-inflammatory action and analgesic effects. All tested compounds had distinct effects on pH-induced ASIC3 current. APETx2 inhibited only transient current, whereas Ugr9-1 and sevanol decreased transient and sustained components of the current. The effect on mice was evaluated after administering an intramuscular injection in the acetic acid writhing pain model and the complete Freund’s adjuvant-induced thermal hyperalgesia/inflammation test. The bell-shaped dependence of the analgesic effect was observed for APETx2 in the acetic acid-induced writhing test, as well as for sevanol and peptide Ugr9-1 in the thermal hyperalgesia test. This dependence could be evidence of the nonspecific action of compounds in high doses. Compounds reducing both components of ASIC3 current produced more significant pain relief than APETx2, which is an effective inhibitor of a transient current only. Therefore, the comparison of the efficacy of ASIC3 inhibitors revealed the importance of ASIC3-sustained currents’ inhibition for promotion of acidosis-related pain relief.

## 1. Introduction

Different types of cells use special molecular sensors on the membrane to detect extracellular pH. The most specialized sensors of the concentration of protons are members of the degenerin-epithelial Na^+^-channel family called acid-sensing ion channels (ASICs) [1]. Four genes (ACCN1-4) encode at least six splice variants of ASIC subunits: ASIC1a, −1b, −2a, 2b, −3, and −4 [2]. In the central nervous system (CNS), they are involved in monitoring of extracellular pH levels during normal neuronal activity and in the development of pathological processes upon stressful conditions. For example, hypoxia of the brain results in increased glycolysis following an accumulation of lactic acid and acidosis that leads to neuronal cell death. The ASIC1a inhibitors produce a neuroprotective effect in the ischemia model caused by hypoxia in mice [3]. During peripheral inflammation, expression levels of ASIC1a and −2a isoforms were increased in the rat spinal dorsal horn [4]. The potential role for ASICs in the pathogenesis of Parkinson’s disease was shown on the 1-methyl-4-phenyl-1, 2, 3, 6-tetrahydropyridine mouse model [5] but further this data were controverted [6].

In the peripheral nervous system and in the tissues of internal organs, ASICs are responsible for the sensitivity to tissue acidosis, cardiac ischemia, corneal damage, inflammation, and local infections [7,8]. ASIC3 channels are mainly represented in the peripheral nervous system, especially in dorsal root ganglion (DRG) neurons [9,10,11], and inhibition of activity of these channels by the selective ligands is considered to be a promising tool for pain relief [12]. 

An inhibition of ASIC3 was reported to be an attractive approach to producing analgesia. A diuretic agent amiloride, a nonspecific blocker of sodium channels, is one of the most well-known molecules possessing a nonspecific inhibitory effect on ASICs [13]. Other nonspecific substances can also influence ASIC3 activity. Nonsteroidal anti-inflammatory drugs, salicylic acid, and diclofenac were found to inhibit ASICs currents directly on sensory neurons and heterologous-expressed ASIC3 channels [14]. The local anaesthetic tetracaine was found to inhibit the transient ASIC3 currents in a concentration-dependent manner with dependence arising from the extracellular pH value. In addition to ASIC3, tetracaine inhibited the ASIC1a and ASIC1b currents [15]. A small-molecule inhibitor, A-317567, blocked ASIC3’s sustained and transient currents and produced an analgesic effect in an inflammatory thermal hyperalgesia model induced by complete Freund’s adjuvant (CFA) in rats [16].

Natural ASIC3 inhibitors were found to produce analgesia in different animal models of pain. Levo-tetrahydropalmatine (l-THP), a bioactive compound from Chinese herbs of the genera *Stephania* and *Corydalis*, decreased the amplitude of proton-gated currents in DRG neurons and inhibited the nociceptive response to intraplantar acetic acid injections in rats [17]. A natural coumarin derivative osthol was reported to block voltage-gated Na^+^-channels and inhibit ASIC3 [18]. Sevanol from thyme inhibited the amplitude of transient and sustained ASIC3 currents and exhibited pronounced analgesic and anti-inflammatory activity in mice at doses of 1–10 mg/kg [19].

Peptide antagonists of ASIC3 channels were isolated from the venom of sea anemones; that is, the toxin APETx2, its close structural homologue Hcr1b-1, and peptide Ugr9-1 [20,21,22]. APETx2 was shown to inhibit the transient component of ASIC3 current and produce potent analgesic effects in several models of pain on rats (acid-induced muscle pain, peripheral inflammatory pain, and postoperative pain) [23,24,25]. Ugr9-1 was shown to inhibit both components of the ASIC3 current and demonstrated a significant analgesic effect in vivo at 0.1–0.5 mg/kg doses [22,26].

To date, many reports support the direct correlation between ASIC3 activation and inflammatory pain. Here, for the first time, we compare the activity of ASIC3 inhibitors in vitro and in vivo under the same conditions. For this purpose, we selected four compounds that act differently on ASIC3 channels: peptide APETx2 inhibiting only a transient component of the current; peptide Ugr9-1 acting on both components of the current; low-molecular weight compound sevanol, which inhibits two components of the current; and diclofenac, a potent nonsteroidal anti-inflammatory drug, as a positive control of analgesic effects and an inhibitor of the sustained component of the current. The comparison of ASIC3-selective ligands in different animal pain models provides an opportunity to estimate their pharmacological potential and specify the properties of the most attractive compound for drug development.

## 2. Results

### 2.1. Whole Cell Electrophysiology

**Figure 1:**
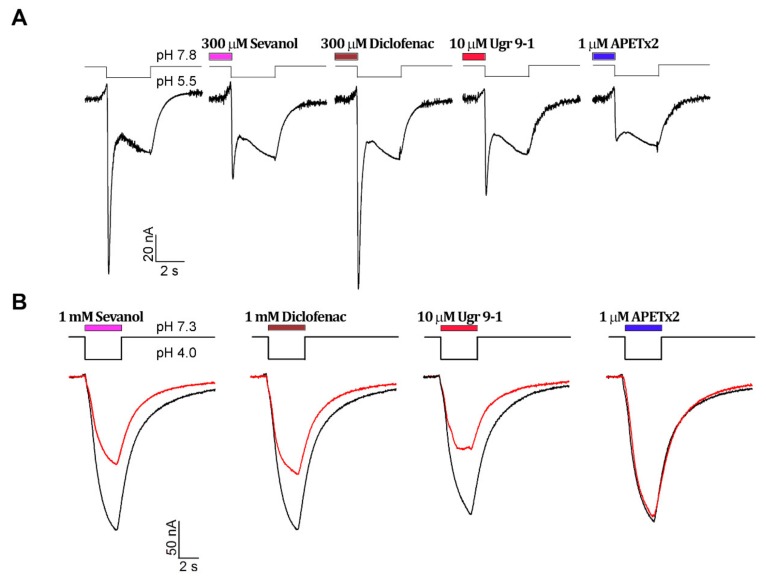

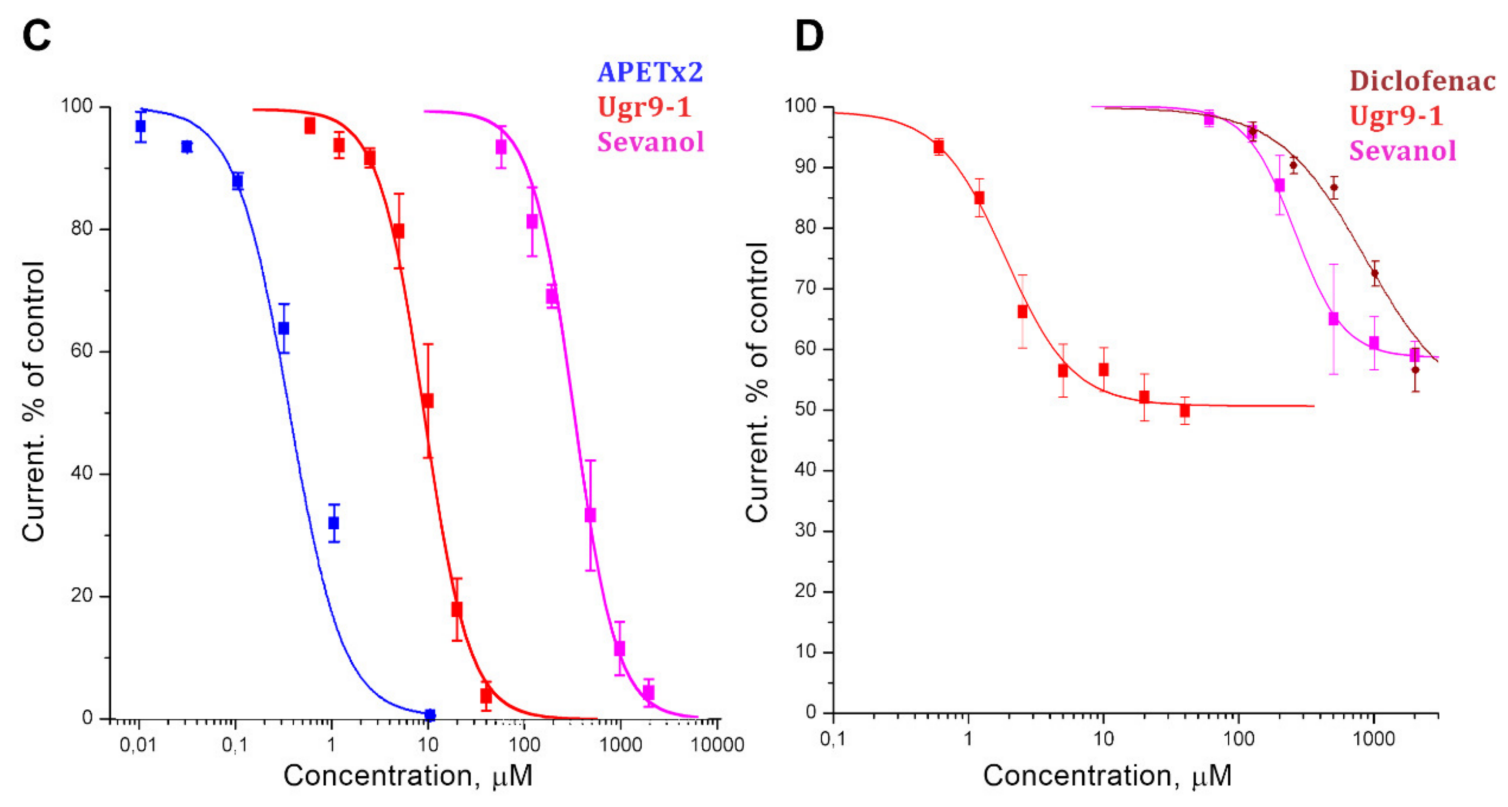
Comparison of ligands’ antagonistic effects on hASIC3 channels. Whole-cell currents were induced by pH drops and recorded at the holding potential −50 mV. (**A**) Effect of ligands on the transient component of current at conditioning pH 7.8. The control trace is shown first; (**B**) Effect of ligands on the sustained component at conditioning pH 7.3. The black line is the control trace, and the red line is the trace of activation in the presence of a ligand. Dose-response curves for transient (**C**) and sustained (**D**) currents’ inhibitions are shown. Data are shown as mean ± SEM (*n* = 4–6) and fitted with the logistic equation (solid lines).

The activity of ligands was checked on human ASIC3 (hASIC3) channels expressed in *Xenopus laevis* oocytes. Sevanol, diclofenac, and two recombinant analogues of peptides (APETx2 and Ugr9-1) were analysed. Two different protocols were used to reveal the effects of ligands on sustained and transient components of acid-induced hASIC3 currents. The influence of the compounds on the transient current amplitude was estimated after preincubation for 15 s before the activation in a low alkali bath solution (pH 7.8), which ensured an absence of steady-state desensitization for the transient current [27] (Figure 1A). The compounds’ inhibition effectiveness was calculated using the value of maximal amplitudes. As expected from previous reports, the APETx2 toxin was the most effective inhibitor of the transient current (Figure 1C). The peptide Ugr9-1 also completely blocked the transient current of ASIC3, but in 30-times greater concentration. Nonpeptide ligand sevanol had much less potency and inhibited transient currents in submillimolar concentrations. Thus, both peptides and sevanol inhibited dose-dependent transient currents at pH 7.8, and the complete inhibition was observed for all of them. The inhibitory effect was concentration-dependent and fit well using a logistic equation. The estimated IC_50_ and Hill coefficient (n_H_) values are summarized in Table 1. The inhibition of transient current by diclofenac was not detected.

The analysis of a sustained component of the current was carried out in a bath solution (pH 7.3) where the transient component of the current was completely desensitized by protons [27]. The pH activation stimulus (3.5 s) was applied in the presence of the testing compound (Figure 1B). APETx2 did not inhibit the sustained current as reported earlier [20]. Sevanol and diclofenac were significantly weaker in their inhibitory potency when compared to peptide toxin Ugr9-1 (Figure 1D, Table 1). The sustained current amplitude was not completely blocked by any of the applied antagonists. The saturation concentrations of ligands (about 3 mM for sevanol and diclofenac and 50 µM for Ugr9-1) inhibited approximately 50% of the sustained current amplitude. The inhibitory effect was concentration-dependent, and the calculated IC_50_ and n_H_ values are summarized in Table 1.

Thus, we evaluated and generally confirmed the pharmacological properties of the compounds inhibiting ASIC3. Tested compounds differ in their abilities to inhibit transient and sustained current components and have activity in broad ranges of concentrations (IC_50_ range 0.3–300 µM for a transient current and 1–800 µM for a sustained current).

### 2.2. Open Field Test

**Figure 2:**
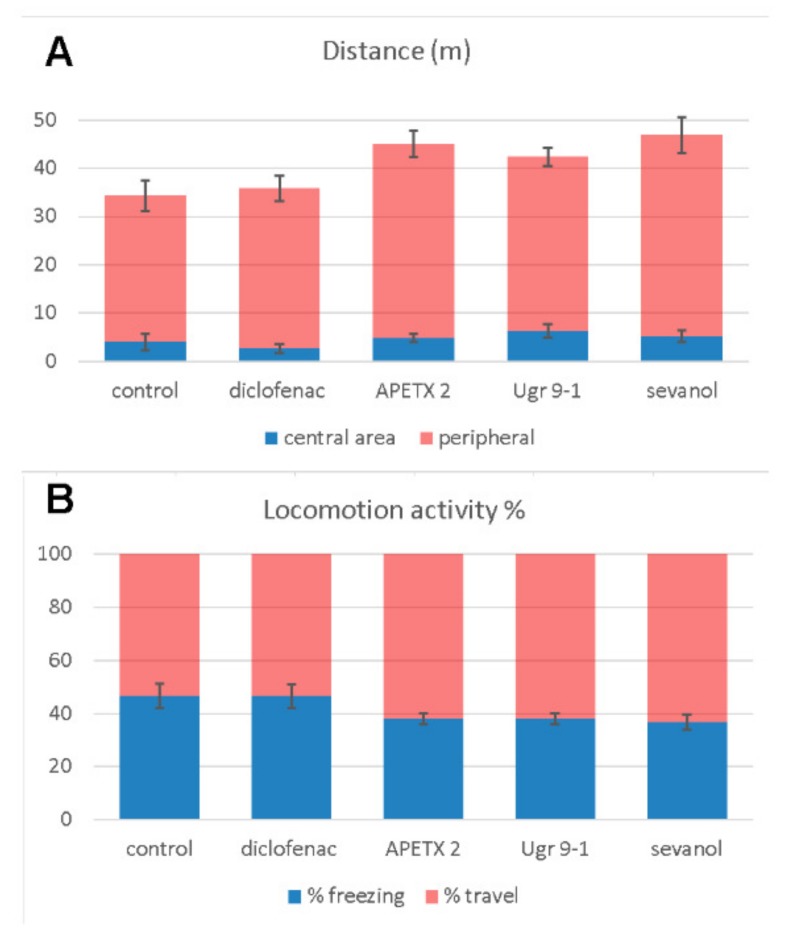
Open field experiments on mice. Administration of compounds at a 1 mg/kg dose intramuscularly was done 4 h before of the measurements. (**A**) Travelling distance and (**B**) locomotion activity presented as mean ± SEM for 5-min recording period (*n* = 6–7).

An open field test was carried out on mice to estimate the possible sedative effects of compounds administration. No statistically significant changes were detected between ASIC3 antagonist-treated groups and the control animals group in 5-min observations of 12 parameters. Animals treated with APETx2, Ugr9-1, and sevanol had a greater travelled distance, a less percentage of time spent freezing (Figure 2A,B) and less travelling distance in the central area, but each of these differences was nonsignificant. Thus, the compounds did not impair locomotion, and the CNS-mediated behaviour and, most likely, their analgesic effects are the result of direct action on peripheral nociceptors.

### 2.3. Acetic Acid-Induced Writhing

**Figure 3:**
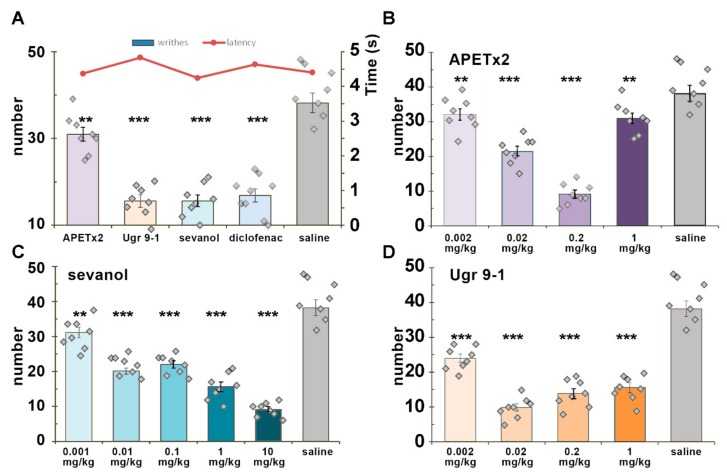
Effects of ligands in an acetic acid-induced writhing test. Pretreatment of mice with APETx2, sevanol, and Ugr9-1 (2 h before testing) attenuated the response to the intraperitoneal administration of acetic acid. (**A**) Efficacy of ASIC3 antagonists at a dose of 1 mg/kg. (**B**–**D**) Dose-dependent chart of ligands’ effects: APETx2 (**B**), sevanol (**C**), and Ugr9-1 (**D**). Results are presented as mean ± SEM (*n* = 8). ** *p* < 0.01, *** *p* < 0.001 versus saline group (ANOVA followed by a Tukey’s test).

Acetic acid-induced writhing is based on irritation of tissues and organs of the abdomen by low pH and could be considered the most appropriate test for influence on acid-induced pain. The dose of 1 mg/kg of all testing compounds was able to reduce the number of writhes significantly and had no influence on the latency time of the first response (Figure 3A). A dose-dependent analysis revealed a bell-shaped profile of APETx2 activity. The APETx2 toxin was surprisingly much less effective at 1 mg/kg than at 0.2 mg/kg dose (19% vs. 76%). The maximal effect was registered for Ugr9-1 at a 0.02 mg/kg dose (74% inhibition) and for APETx2 at a 0.2 mg/kg dose (76% inhibition) (Figure 3B,D). The plant lignan sevanol showed a linear dose dependence with a maximal effect at a 10 mg/kg dose (76% inhibition). It is interesting that the effects of sevanol and Ugr9-1 plateaued at a wide range of doses (0.01–1mg/kg for sevanol and 0.02–1 mg/kg Ugr9-1) (Figure 3C,D).

### 2.4. CFA-Induced Inflammation

**Figure 4:**
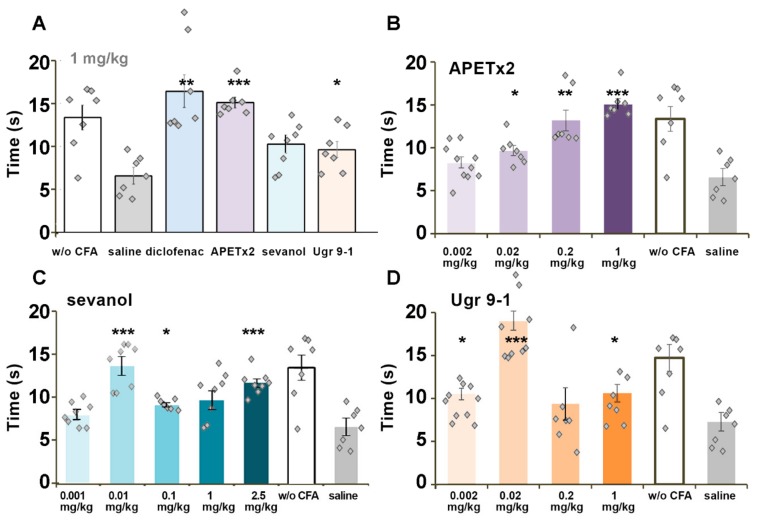
Effect of ligands on the CFA-induced thermal hyperalgesia test. Test was performed 2 h after intramuscular administration of ASIC3 antagonists. (**A**) Comparison between ligands at the dose of 1 mg/kg; (**B**–**D**) Dose-dependent chart of ligands’ effect. APETx2 (**B**), sevanol (**C**), and Ugr9-1 (**D**) reversed CFA-induced thermal hyperalgesia and prolonging withdrawal latency of the inflamed hind paw on a hot plate. Results are presented as mean ± SEM (*n* = 7–8). * *p* < 0.05, ** *p* < 0.01, *** *p* < 0.001 versus saline group (ANOVA followed by a Tukey’s test).

CFA-induced thermal hyperalgesia is a result of different inflammatory pathways’ action on thermal sensitivity of the paw. Injection of CFA into the hind paw provokes increased sensitivity to noxious mechanical and thermal stimuli together with swelling of the paw due to the inflammatory process. Diclofenac and APETx2 at 1 mg/kg doses showed equal potency in the reversal of thermal hyperalgesia, which exceeded the analgesic potency of sevanol and Ugr9-1 at the same dosage (Figure 4A). For APETx2 (Figure 4B), sevanol (Figure 4C), and Ugr9-1 (Figure 4D), the dose dependency of the analgesic effect was evaluated. The effect of APETx2 was dose-dependent with a minimal active dose of 0.02 mg/kg (47% of reversal), and the complete reversal of thermal hyperalgesia was reached at doses of more than 0.2 mg/kg. The effects of sevanol and Ugr9-1 were bell-shaped, so the reversal of thermal hyperalgesia for 1 mg/kg and 0.001–0.002 mg/kg doses did not differ significantly (Figure 4C,D). The maximal effect value for both samples was observed in doses of 0.01–0.02 mg/kg. Additionally, the effect of sevanol at 0.01 mg/kg and Ugr9-1 at 0.02 mg/kg was confirmed in the independent experiment (data not shown).

**Figure 5:**
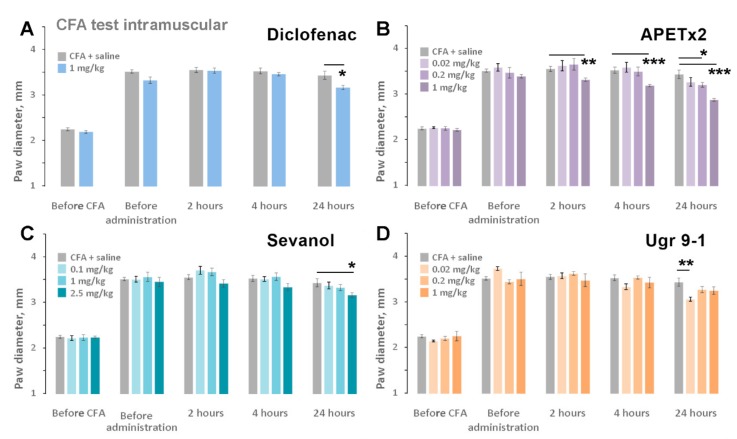
Anti-inflammatory effect of ligands. Paw oedema induced by CFA injection was estimated before the CFA and testing compounds administration and 2, 4, and 24 h after the intramuscular injection of diclofenac (**A**), APETx2 (**B**), sevanol (**C**), and Ugr9-1 (**D**). Results are presented as mean ± SEM (*n* = 7–8). * *p* < 0.05, ** *p* < 0.01, *** *p* < 0.001 versus saline group (ANOVA followed by a Tukey’s test).

To reveal the anti-inflammatory effects of ASIC3 antagonists, paw oedema changes were also evaluated (Figure 5). Diclofenac, sevanol, and Ugr9-1 did not produce significant anti-inflammatory effects within 4 h after administration. APETx2 in a dose of 1 mg/kg reduced paw oedema by 16%, 24%, and 44% compared to the control group at 2, 4, and 24 h after administration, respectively. APETx2 at 0.2 mg/kg reduced inflammation by approximately 20% only 24 h after administration. Peptide Ugr9-1 (0.02 mg/kg) and sevanol (2.5 mg/kg) also reduced the inflammation process within 24 h by 31% and 22%, respectively.

## 3. Discussion

ASIC3 channels play an important role in the perception and maintenance of pain signals in peripheral neurons. Several ligands with inhibitory properties were found for this receptor, each of which was able to induce analgesia in animal tests. The most well-known inhibitor APETx2 in various studies has been tested on models of acid-induced muscle pain model, CFA-induced inflammatory pain model (at doses of 0.07, 0.22 and 2.2 µM) [25]. Ugr9-1 was studied in models of CFA-induced thermal hyperalgesia and acetic acid writhing test (at doses of 0.5, 0.1, and 0.01 mg/kg) [22]. Sevanol was studied in CFA-induced thermal hyperalgesia and in response to intraperitoneal administration of acetic acid (at doses of 1 and 10 mg/kg) [19]. Diclofenac significantly reduced CFA-induced thermal hyperalgesia (100 mg/kg i.p.) [28] and inhibited acetic acid-induced writhing (20 mg/kg i.p.) [29]. Therefore, it was extremely interesting to compare these ligands together in the same pain models. A comparison between the antagonists acting on both components of the ASIC3 current and APETx2 inhibiting only the transient current was important for the understanding of their contributions to pain perception. 

APETx2 is the most well-studied and well-known inhibitor of ASIC3 channels despite proven inhibitory activity on Na_v_1.2 (IC_50_ ~ 113 nM) and Na_v_1.8 channels (IC_50_ ~55 nM on *X. laevis* oocytes and ~2.6 µM on DRG neurones) [30,31] and the inhibition of hERG channels by reducing the maximal current amplitude and shifting the voltage dependence of activation to more positive potentials [32]. Moreover, APETx2 potentiates rat ASIC1b and ASIC2a at concentrations of 3–10 µM (30–100-fold higher than its ASIC3 inhibitory concentration), which may have implications for its use in in vivo experiments [33]. The possible cumulative effect on pain relief via ASICs and Na_v_1.8 channel inhibition increases the apparent efficacy, but masks the real significance of the acid-sensing pathway inhibition in behavioural tests. Moreover, potentiating ASIC1b most likely counteracts the antihyperalgesic effects produced by ASIC3 inhibition [33]. 

The importance of the ASIC3 channel in nociception was proven by using APETx2 in several animal models of pain. This toxin produced pain relief in bone, tooth, muscle, and skin pain as well as in gastric acidosis and gastric mucosal lesions, osteoarthritis inflammation, fibromyalgia-induced mechanical hyperalgesia, fatigue-enhanced hyperalgesia, and postoperative hyperalgesia [34,35,36,37,38,39,40,41].

Here, we found that in vitro APETx2 was significantly better (IC_50_ was 344 ± 80 nM) at inhibiting the transient current of hASIC3 than the other tested antagonists (Figure 1). The activity of recombinant APETx2 on hASIC3 in the oocyte system could be considered equipotent to the published data, where toxin inhibited human ASIC3 and rat ASIC3 expressed in COS cells (**C**V-1 (simian) in **O**rigin, and carrying the **S**V40 genetic material) with the IC_50_ of 175 nM and 63 nM, respectively [20]. Peptide Ugr9-1 and sevanol had the same inhibitory effects as reported earlier [19,22]. Therefore, in vitro potency of compounds to inhibit the transient component of hASIC3 current could be ranged as APETx2 > Ugr9-1 > sevanol. The potency of sustained component inhibition was ranged as Ugr9-1 > sevanol > diclofenac. 

Dose-response analysis was used to acquire a clear relationship between a dose and the extent of the response to it. However, in various tests diverse compounds showed so-called “bell-shaped” or “(inverse) U-shaped” dose response. The best example of this effect was the dose dependence of the analgesic effect of APETx2 in the acetic acid writhing test (Figure 3B). The analgesic effect evidently increased up to 75% at 0.2 mg/kg dose, whereas the peptide was significantly less effective at a 1 mg/kg. Many researchers have described this phenomenon [42,43]. This type of dose response was described for micronutrients, endocrine-disrupting chemicals, endogenous hormones, and other drugs. 

Several possible mechanisms of this phenomenon were suggested: a high-dose induction of cytotoxicity; the activity of ligand on different receptors or receptors’ states depending on concentration following the competition between multiple receptor pathways; and ligand-induced receptor down-regulation at high concentrations. Tested compounds were studied in electrophysiological experiments on *X. laevis* oocytes and Chinese hamster ovary (CHO) cells in various concentrations, and no cytolytic or cytotoxic effects were observed. Therefore, a high concentration of ASIC3 ligand is most likely the result of activity on other targets and/or an unfavourable breakdown of the ASIC3 signalling pathway (e.g., ligand-induced receptor down-regulation and compensation by other receptors). APETx2 at high concentrations is also able to potentiate ASIC1b, which is involved in peripheral nociception in rodents. Many examples demonstrate how positive modulation of the receptor could dramatically change the overall effect of the substance. Potentiation or weak activation of TRPA1 (pain receptor responsible for the detection of harmful chemicals) can surprisingly cause strong analgesia instead of pain [44,45,46]. Another example, potentiation of TRPV1 weak activation, gives sea anemone peptides APHC1 and APHC3 (inhibitors of TRPV1) an ability to decrease core body temperature in mice, whereas most of the other inhibitors of TRPV1 cause hyperthermia [47,48]. 

When the pain was induced by an acetic acid injection (acetic acid-induced writhing test), all of the ASIC3 antagonists, including diclofenac, showed high efficacy and the same maximal effect of reducing pain (~75%). The most stable effect was produced by Ugr9-1, and it was slightly more significant in lower doses (0.002 mg/kg). The efficacy of sevanol was also high. Despite the 1000-fold difference in efficacy in vitro with APETx2 and the 35-fold difference with Ugr9-1, sevanol produced an almost equipotent effect in vivo (molecular weight ratio APETx2/sevanol is 6.4 and Ugr9-1/sevanol is 4.4). However, sevanol is also able to inhibit ASIC1a [19] and produce additional analgesic effect. Blockade of ASIC1a in CNS by psalmotoxin 1 was reported to result in an activation of the endogenous enkephalin pathway and potent analgesic effects [49]. Therefore, it is better to compare the effectiveness of peptides Ugr9-1 and APETx2 for contributions of sustained and transient current in the perception of acid-induced pain in the peripheral nervous system. Peptides are usually unable to penetrate through the blood-brain barrier, therefore no CNS targets could be suspected in additional effects. Ugr9-1 had an effective dose 10-times less than APETx2 in acetic acid-induced writhing test pain, whereas it was much less effective (30 times) in inhibition of the transient component of ASIC3 current. Therefore, we can conclude that a sustained component can make a significant contribution to the development of acid-induced pain, and compounds that inhibit both components of ASIC3 channels are more effective for the treatment of acidosis-associated pain conditions.

In the acetic acid-induced writhing test, only APETx2 showed “U-shaped” dose response with maximal effect at 0.2 mg/kg dose. The decrease of effect at a high dose is most likely the result of conflict between inhibition of ASIC3 and enhanced activation of ASIC1b. 

CFA-induced inflammation is a general model of inflammatory pain. The TRPV1 receptor is considered to play a leading role in thermal hyperalgesia induced by CFA [50], which could be modulated via other receptors including ASIC3 [51]. All ASIC3 antagonists were capable of completely reversing thermal hyperalgesia. The APETx2 peptide dose-dependently reversed thermal hyperalgesia and reached its maximal effect at 0.2 mg/kg, which corresponds well with previously reported results [24,25]. Despite a great number of described side targets, it is noteworthy that APETx2 showed standard dose response.

Sevanol and Ugr9-1 were more potent in reaching maximal effects in the hyperalgesia tests at doses of 0.01 and 0.02 mg/kg versus the 0.2 mg/kg dose for APETx2. In this test, sevanol and Ugr9-1 showed a nonlinear dose response. Sevanol was unexpectedly more active at a lower dose than peptide Ugr9-1, whereas the peptide showed better inhibition of ASIC3 in vitro. Several explanations could be proposed. First, the result of partial ASIC1a inhibition is unpredictable. Second, better bioavailability of sevanol compared with Ugr9-1 can enhance the effect. Peptides from sea anemones have rather short circulating half-lives (~0.5 h) after subcutaneous or intramuscular administration but could circulate in mammals’ blood flow for a long time (over 72 h) before complete elimination [52,53]. It was proposed that the significant part of a peptide could be cleared out from blood by kidneys, whereas a small fraction distributes into some compartment from which the active peptide is released slowly into the blood stream [52]. The low molecular weight compound diclofenac has exponential decrease of concentration to zero with circulating half-life of about 2 h and finally eliminates from the blood flow at 10 h [54]. The stability of sevanol in blood was most likely greater than the rate of elimination/deponation of Ugr9-1. Therefore, a dose of 0.01 mg/kg of sevanol could produce the same analgesic effect in a CFA test as 0.02 mg/kg of Ugr9-1. The absence of the effect at doses 0.001 mg/kg and 0.002 mg/kg for sevanol and Ugr9-1 could be the result of the quick elimination of low doses of compounds.

Therefore, the inhibition of the sustained current should be considered as a major benefit providing effectiveness of sevanol and Ugr9-1 at low doses, but their efficacy significantly decreased at higher doses; the reasons for this remain unclear. The nonspecific actions on other cellular targets and the changes of ASIC3 involvement in the inflammatory process induced by the high antagonists’ concentration for 2 h are suspected. We conclude that the inhibition of both components of ASIC3 currents could be beneficial but could be reversed by hormesis in high doses and during a long exposure to an ASIC3 antagonist. The efficacy and final effects of ASIC3 antagonists most likely depend on the route of administration and the time interval before testing. For example, when injected intravenously 30 min before testing, Ugr9-1 was clearly dose-dependent in the CFA thermal hyperalgesia test and showed less efficacy in the acetic writhing test (~50%) [22].

To date, various studies have shown the important role of the sustained component of ASIC3 in inflammatory conditions [24,55,56]. Because a significant qualitative difference between sevanol and Ugr-9-1 versus APETx2 is the inhibition of the sustained component, we have suggested that the more effective anti-inflammatory effect of sevanol and Ugr-9-1 may be a result of sustained component inhibition.

All of the tested compounds displayed poor anti-inflammatory activity. This is most likely because ASIC3 activation plays a significant role only in late phases of paw oedema development [57]. This is well correlated with the observed oedema reduction during the 24-h activity lag. The 1 mg/kg dose of APETx2 was effective 2 h after the injection; however, the effect could be associated with the inhibition of Na_V_1.2 and 1.8 channels and not a direct action on ASIC3.

Finally, we can assume that all tested antagonists for ASIC3 showed high efficacy in animal models of pain, but could have bell-shaped dose response in some tests in vivo. The higher potency of APETx2 in inhibiting transient currents of ASIC3 gave no advantages in analgesic activity compared to sevanol and Ugr9-1 (inhibitors of both transient and sustained currents) as revealed in the two pain models (acetic acid-induced writhing test and thermal hyperalgesia). The reasons for the bell-shaped dose response of sevanol and Ugr9-1 remain unclear, but this effect should be taken into consideration while testing novel ASIC3 antagonists.

## 4. Materials and Methods 

### 4.1. Ligands

Peptide Ugr9-1 was obtained by the production of a recombinant analogue in *Escherichia coli* (as described in [22]). Peptide APETx2 was also produced by the heterologous expression in *E. coli* [58]. Synthetic sevanol was obtained from the Laboratory of Biopharmaceutics of the Shemyakin-Ovchinnikov Institute of Bioorganic Chemistry of the Russian Academy of Sciences. Diclofenac was purchased from (Sigma-Aldrich, Moscow, Russia).

### 4.2. Electrophysiology

*X. laevis* oocytes were removed surgically and defolliculated by collagenase (Sigma-Aldrich, Moscow, Russia). Oocytes were injected with 2.5 to 10 ng of human ASIC3 cRNA (AF057711.1). The cRNA transcripts were synthesised from a NaeI-linearized ASIC3 cDNA template (pcDNA3.1+humanASIC3 subcloned from clone EX-Q0260- B02 (GeneCopoeia, Inc., Rockville, MD USA) using a HiScribe T7 High Yield RNA Synthesis Kit (New England Biolabs, Ipswich, MA, USA) according to the manufacturer’s protocol for capped transcripts. After the injection, oocytes were kept for 3 to 6 days at 19° in a ND-96 medium containing (in mM) 96 NaCl, 2 KCl, 1.8 CaCl_2_, 1 MgCl_2_, and 5 HEPES titrated to pH 7.4 with NaOH supplemented with gentamycin (50 µg/mL). Two-electrode voltage clamp recordings were performed using a GeneClamp 500 amplifier (Axon Instruments, Union City, CA, USA), and the data were filtered at 20 Hz and digitized at 100 Hz by an AD converter L780 (LCard, Moscow, Russia) using in-house software. To induce transient and/or sustained currents, two different protocols with different conditioning pH were used. Microelectrodes were filled with a 3 M KCl solution. The working buffer solution was ND-96 titrated by NaOH to pH 7.8 or 7.3. The solution for the pH shift was constructed based on the ND-96 solution, in which 5 mM HEPES was replaced with 10 mM acetic acid (pH 4.0) or 10 mM MES (pH 5.5) in a supplementary 0.1% BSA solution. The duration of the activation pulses was 3.5 s.

### 4.3. Animals

Adult male CD-1 mice (Animal Breeding Facility Branch of Shemyakin-Ovchinnikov Institute of Bioorganic Chemistry, Russian Academy of Sciences, Pushchino, Russia) weighing 20–25 g were used. Animals that originally passed clinical examination that confirmed no deviations in health were divided into groups (at least eight male mice per group) using the principle of randomization. The average body weight of animals in each group was not statistically different between groups. Animals were housed at room temperature (23 ± 2 °C) and subjected to a 12-h light-dark cycle with food and water available ad libitum. All experiments were performed after receiving approval from the Animal Care and Use Committee of the Branch of the Institute of Bioorganic Chemistry, Russian Academy of Sciences (IBCh RAS) (Pushchino, Russia Federation). The samples were administered intramuscularly 2 h before testing (4 h in the open field test). The initial dose of 1 mg/kg was investigated for all samples, which was further reduced 5, 50, and 500 times for peptides. In the case of sevanol as the least active molecule in vitro, this dose was not only reduced 10, 100, and 1000 times, but was increased to 2.5 or 10 mg/kg in some experiments.

#### 4.3.1. CFA-Induced Thermal Hyperalgesia

CFA suspended in an oil/saline (1:1) emulsion was injected into the dorsal surfaces of the left hind paws of mice (20 µL/paw) 24 h before the samples’ intramuscular injection. The control mice received 20 µL of saline (intraplantar). The paw withdrawal latencies to thermal stimulation (53 °C) were measured 2 h after the sample injection. The paw diameter was evaluated before the CFA injection, before the samples and saline administration, and 2, 4, and 24 h after the administration using a digital calliper.

#### 4.3.2. Acetic Acid-Induced Writhing (Abdominal Constriction Test of Visceral Pain)

Separate groups of mice were used. Writhes were caused 2 h after the intramuscular injection of the samples (saline for control mice) with the injection of 0.6% acetic acid in saline (10 mL/kg intraperitoneally). Mice were immediately placed inside transparent glass cylinders, and the latency of a first writhe and the number of writhes were recorded for 15 min. 

#### 4.3.3. Open Field Test

In the locomotion measurements, exploration and anxiety were evaluated in the motor activity test on a computerized TSE Multi Conditioning System (TSE Systems GmbH, Bad Homburg, Germany). Four hours after the administration of the sample, the measurement was made, which lasted for 3 min. The animal watching TSE AatiMot programme (TSE Systems GmbH, Bad Homburg, Germany) was used to collect and analyse the control parameters.

### 4.4. Data Analysis

The analysis of the electrophysiological data was performed using the OriginPro 8.6 programme. The four parameter logistic equation was used for concentration–response curves: F(x) = ((a1 − a2)/(1 + (x/x0)n)) + a2, where x is the concentration of sample; F(x) is the response value at given sample concentration; a1 is the control response value (fixed at 100%); x0 is the IC_50_ value; n is the Hill coefficient (slope factor); and a2 is the response value at maximal inhibition (percent of control).

The data significance in animal tests was determined by an analysis of variance followed by a Tukey’s test. Data are presented as the mean ± SEM.

## Figures and Tables

**Table 1:** Inhibition potency of hASIC3 antagonists.

Antagonist	Transient Currents		Sustained Currents	
	IC_50_ (µM)	Hill Coefficient	IC_50_ (µM)	Hill Coefficient
APETx2	0.344 ± 0.080	1.5 ± 0.2	-	-
Ugr 9-1	9.1 ± 0.9	1.86 ± 0.16	1.88 ± 0.36	1.74 ± 0.51
sevanol	331 ± 21	1.98 ± 0.24	259 ± 39	2.4 ± 0.4
diclofenac	-	-	856 ± 106	1.35 ± 0.21
